# A Novel Fixation Method for Variable-Sized Endoscopic Submucosal Dissection Specimens: An *In Vitro* Animal Experiment

**DOI:** 10.1371/journal.pone.0146573

**Published:** 2016-01-07

**Authors:** Seung Han Kim, Hyuk Soon Choi, Hoon Jai Chun, In Kyung Yoo, Jae Min Lee, Eun Sun Kim, Bora Keum, Yeon Seok Seo, Yoon Tae Jeen, Hong Sik Lee, Soon Ho Um, Chang Duck Kim

**Affiliations:** Division of Gastroenterology and Hepatology, Department of Internal Medicine, Institute of Gastrointestinal Medical Instrument Research, Korea University College of Medicine, Seoul, Republic of Korea; The University of Texas MD Anderson Cancer Center, UNITED STATES

## Abstract

**Background:**

Endoscopic submucosal dissection is considered a curative and minimally invasive treatment for early gastric cancer; however, precise pathologic assessment of resected specimens is required to develop further treatment plans. Human error during specimen handling can affect objective assessment of resected specimens. In this study, we investigated whether a novel tissue fixation device offered more objective and standardized pathologic evaluation than conventional manual tissue fixation.

**Methods:**

We developed a novel tissue fixation device for endoscopic submucosal dissection specimens. Two circular tissue samples 2, 3, and 4 cm in diameter were obtained from the body of 45 porcine stomachs. One specimen sample was placed in a fixation device; the other was manually fixed on corkboard. We used a pressure indicator to ensure constant pressure in the resected specimens in the fixation device. We measured submucosal diameter and thickness after 24 hr.

**Results:**

The diameters for 2, 3, and 4 cm resected tissue samples were 23.85, 32.30, and 45.0 mm and 21.0, 32.0, and 44.50 mm for the fixation device and manual pinning groups, respectively. The submucosal thicknesses in the fixation device group were 397.09, 381.43, and 415.51 μm and 393.76, 529.69, and 603.82 μm by manual pinning for 2, 3, and 4 cm tissue samples, respectively. Analysis of standard deviation revealed that the submucosal thickness in the manual fixation group was much more variable than in the fixation device group (p = 0.012, 0.042, and 0.001 for 2, 3, and 4 cm tissue specimens, respectively; Fligner-Killeen test of homogeneity of variances).

**Conclusions:**

Among variously sized resected tissue specimens, submucosal thicknesses were more variable in the conventional fixation group, while the thicknesses were comparatively consistent in the fixation device group. After endoscopic submucosal dissection, pathologic preparation using this fixation device could offer more objective assessment of specimens.

## Introduction

Endoscopic submucosal dissection (ESD) is considered a curative and minimally invasive early gastric cancer (EGC) treatment [1,2]. Advances in endoscopic techniques permit broader resection of the mucosal and submucosal layers and have become established for wider indications, including a maximum submucosal invasion depth of 500 μm and a diameter up to 3 cm [3–5]. Although ESD for EGC is a safe and curative procedure, the main variables associated with cure are the ability to perform a complete resection and lymph node metastasis [6]. The expanded criteria for curative endoscopic treatment of EGC have been determined after investigation of a large cohort presenting negligible risk of EGC lymph node metastasis based on these criteria. However, the validity of these indications has been debated because of the possibility of unexpected lymph node metastases for EGC treated according to these criteria.

Because lymph node metastasis cannot be assessed before ESD, pathologic assessment of ESD specimens for pathological prognostic risk factors for lymph node metastasis such as the submucosal invasion depth, histological differentiation, lesion size, lymphatic and vessel invasion, and deep and lateral margins, are important to determine patient prognosis and to plan future treatment [4,6].

Precise pathologic assessment of resected cancer tissue is very important; however, recently reported cases of regional lymph node metastasis despite curative endoscopic procedures for EGC based on the expanded ESD criteria [7,8] have raised controversies over the validity of these criteria.

There are several possible explanations for these observations; first, the depth of submucosal invasion, a component of ESD indication, could vary due to human error during specimen handling [9,10]. As resected ESD tissue has considerable elasticity, stretching during handling frequently results in variability in the submucosal invasion depth and tissue diameter. These factors reduce the reliability of micrometric pathologic assessment of resected specimens and may also affect cut-off values of submucosal invasion depth. This problem deserves further investigation because of increasing interest in non-invasive approaches to early gastrointestinal cancer.

To improve patient prognosis after ESD for EGC, more objective and standardized pathologic assessments are required. We aimed to create a novel tissue fixation device that minimalizes the stretching effect and to validate this device by comparing the variability of submucosal thickness and tissue diameter to conventional manual corkboard pinning.

## Materials and Methods

### Sample preparation

**Fig 1:**
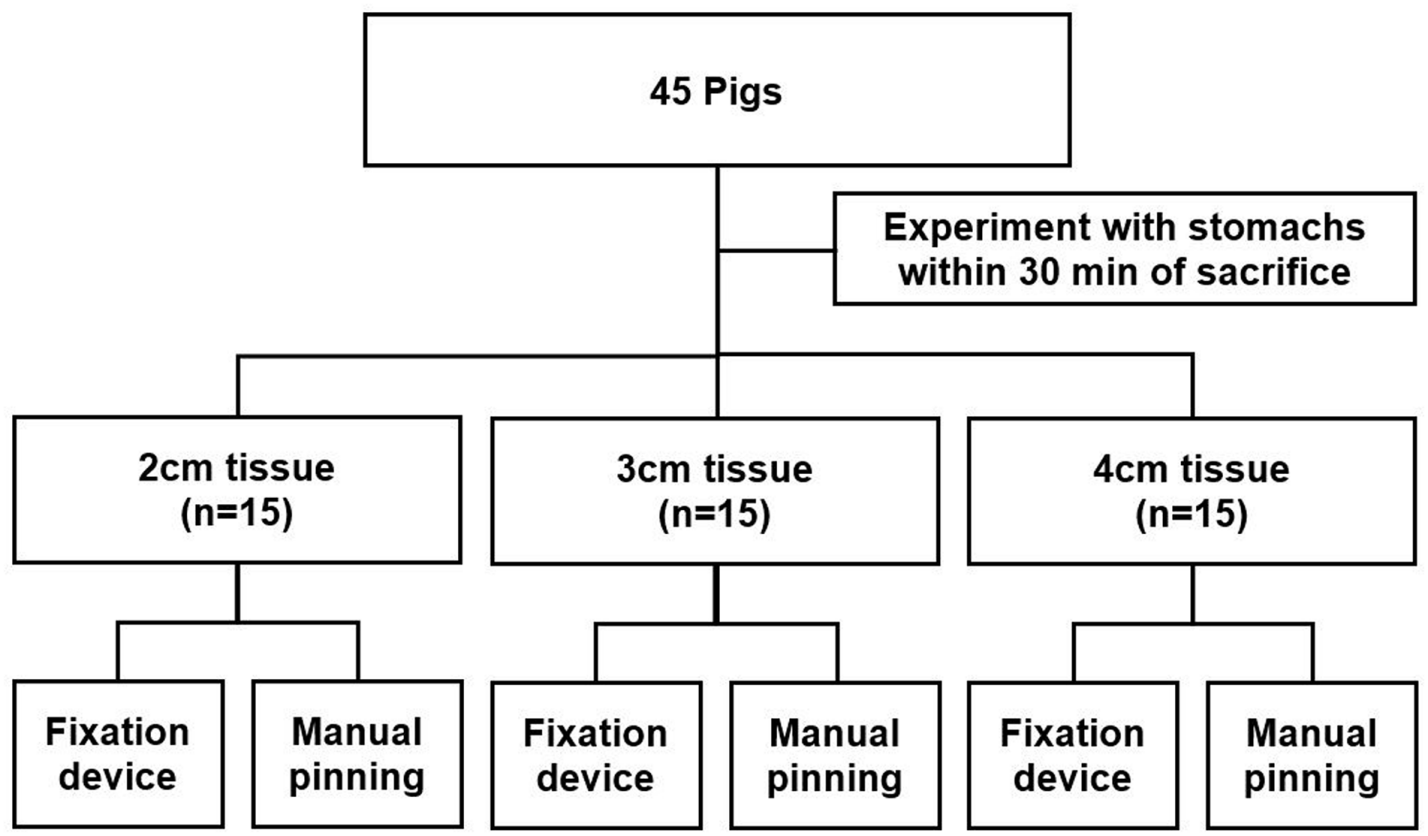
Study flow chart. Forty-five porcine stomachs were used in this study. Fifteen were assigned to each group according to resected tissue size. After resection, one resected specimen each was placed in a fixation device and manually pinned to a corkboard.

From a local butcher shop, we obtained a total of 45 stomachs from pigs weighing 40 to 50 kg. Within 30 min of sacrifice, we resected tissue to obtain two identically sized porcine stomach specimens. All tissue samples were obtained from the body of porcine stomachs. We divided the resected specimens into three groups according to diameter, and split each tissue diameter group into fixation device and manual pinning groups (Fig 1).

### Ethics statement

This study was carried out in strict accordance with the recommendations in the Guide for the Care and Use of Laboratory Animals of the National Institutes of Health. The protocol was approved by the Committee on the Ethics of Animal Experiments of the Korea University Anam Hospital (Permit Number: KUIACUC-2015-251). All efforts were made to minimize suffering.

### Tissue fixation device

**Fig 2:**
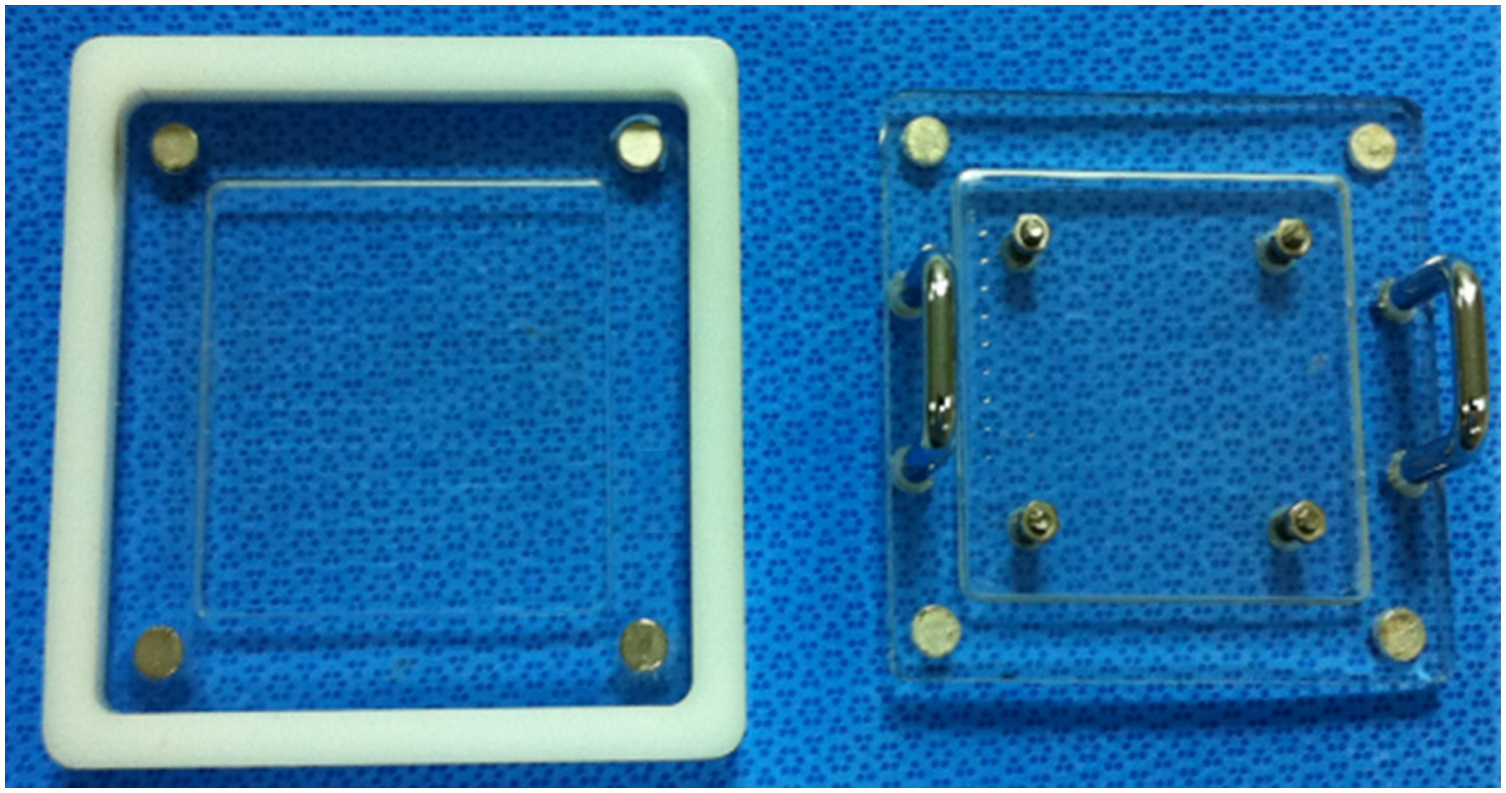
Tissue fixation device. The fixation device was developed to ensure constant tissue elasticity, this prevented tissue deformation during preparation.

In this study, we developed and evaluated a tissue fixation device (S1 Video). Tissue samples were inserted into a 6 cm space, with a magnet attached to the corner making contact with the cover. A spring was attached to the middle to allow flexibility in pressing variable depths of tissue samples (Fig 2). Each spring was associated with four screws that could adjust the pressure of the tissue. Tissues were subjected to slight pressure in order to remove voids between the surface of the sample and the movable top plate. Also, the deformation of the tissues could be reduced in spite of various mucosal depth.

### Porcine stomach resection

**Fig 3:**
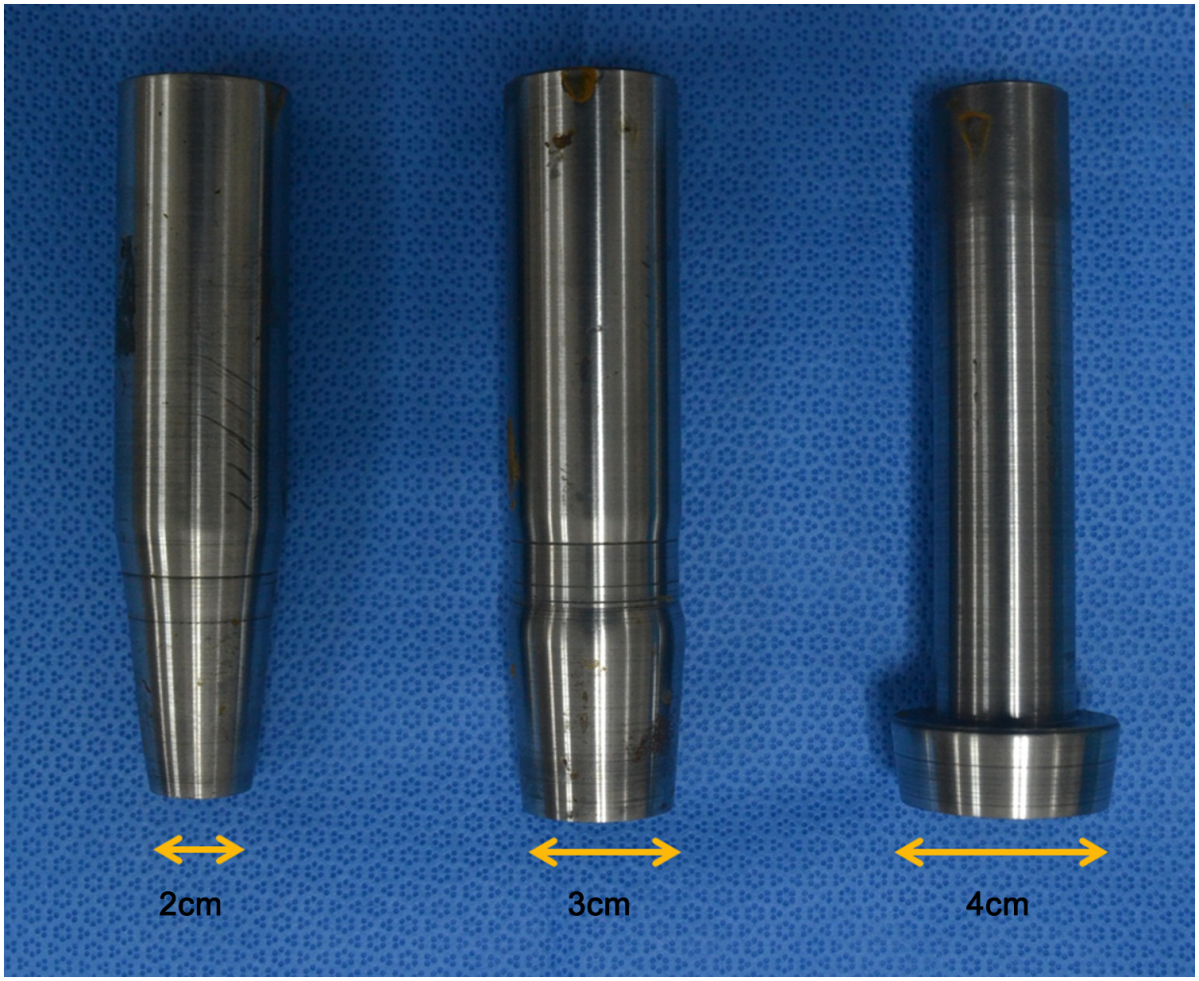
Punch devices according to specimen diameter. All specimens were resected using the punch device.

**Fig 4:**
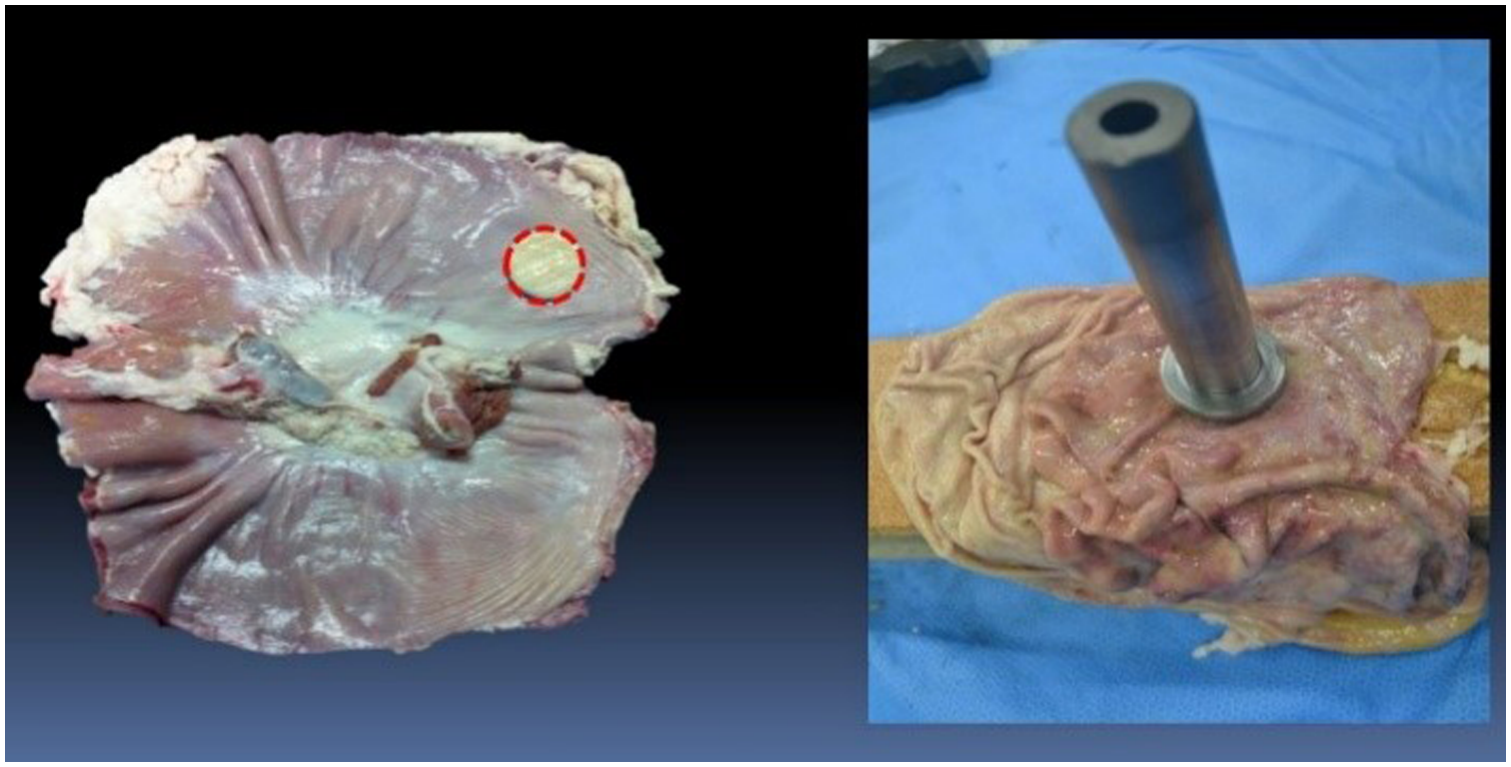
Porcine stomach resection. All specimens were resected from identical porcine stomach lesions using the punch device.

We used punch devices for consistent tissue sampling. Circular specimens 2, 3, and 4 cm in diameter were resected using these punch devices (Figs 3 and 4).

### Degree of Pressure

**Fig 5:**
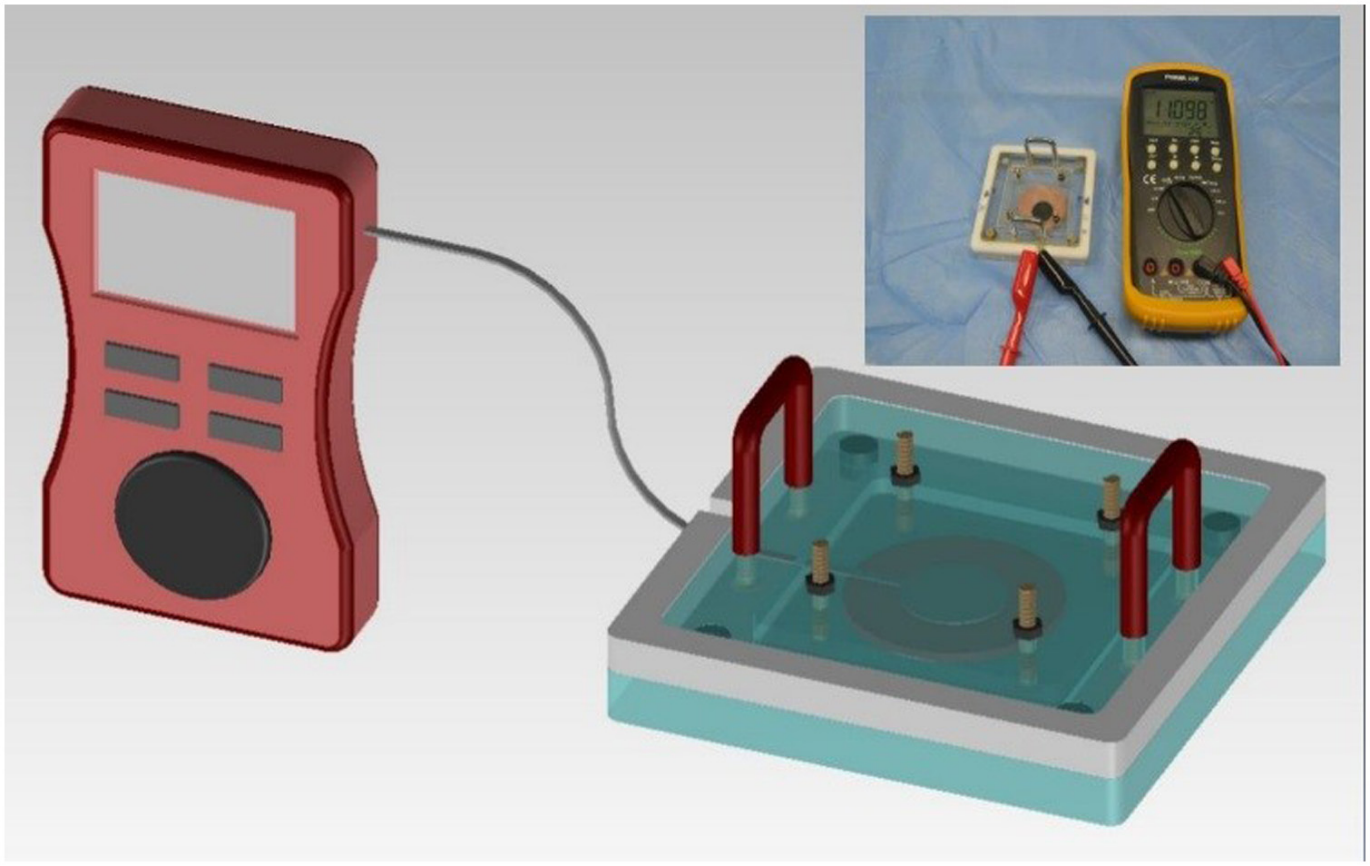
Pressure indicator. Using the pressure indicator, the tissue samples were maintained under a constant pressure.

A pressure indicator was used to maintain constant pressure on the tissue specimens. The pressure on tissue harvested from the stomachs used in this study was measured as shown in Fig 5. Tissue samples were mounted between the moving and fixed plates in the device. After placing the tissues in the fixation device, a constant pressure was applied. We applied pressure up to a reference value that was constant when the voids between the surface of the sample and the top plate were eliminated.

### Manual Pinning

Manual pinning was performed as follows. First, four physicians performed manual pinning of each tissue specimen to compare inter-evaluator variation. Second, we attempted to avoid curling of the edges during fixation. Third, we fixed specimens with pins or thin needles. All tissues were handled gently to avoid tearing the tissue.

### Comparison of Fixation Methods

**Fig 6:**
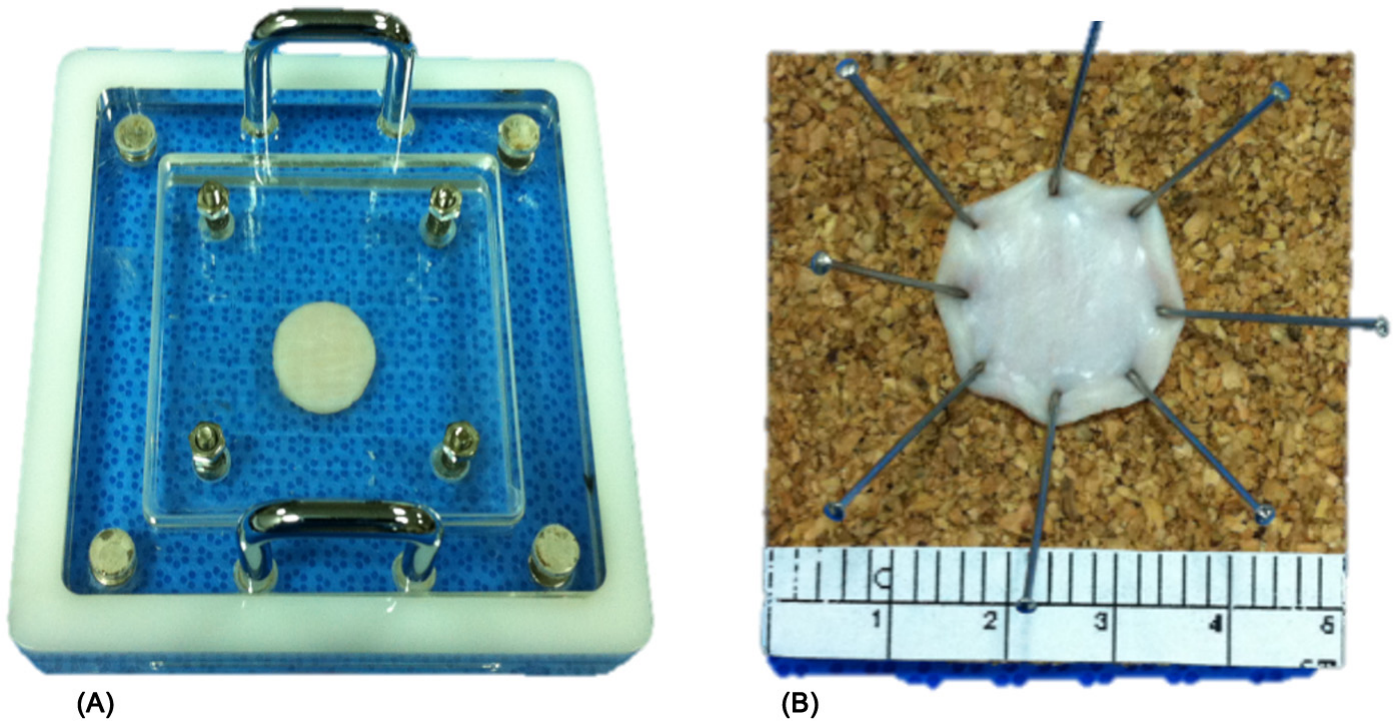
Two tissue fixation methods (A) fixation device and (B) manual pinning. After being resected from porcine stomachs, the specimens were prepared using two tissue fixation methods. (A) One specimen was placed in the fixation device; (B) a second specimen was manually fixed to a corkboard.

We compared two methods of tissue fixation: one piece of tissue was inserted in a fixation device and the other was manually pinned on corkboard. After formalin fixation for 24 h, we measured the submucosal thickness and tissue diameter (Fig 6).

### Microscopic Evaluation

**Fig 7:**
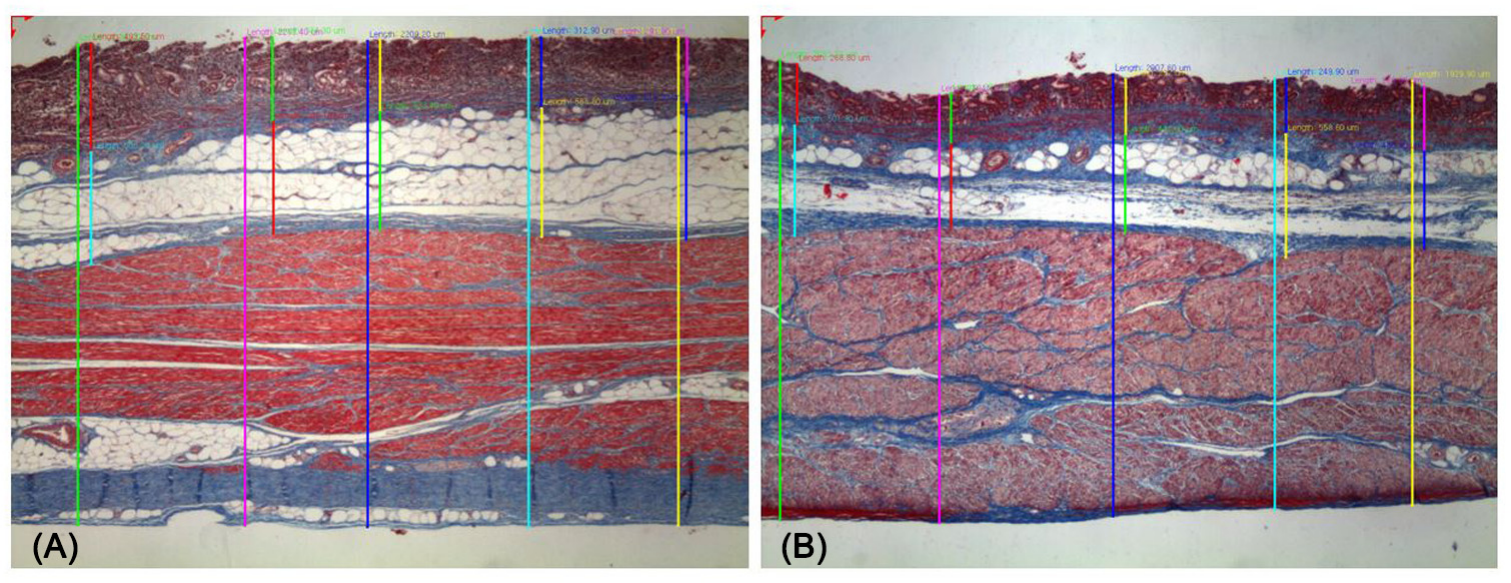
Endoscopic submucosal dissection specimens processed by two fixation methods. (A) One specimen processed by fixation device (Masson-trichrome stain, ×20); (B) a second specimen processed by manual pinning (Masson-trichrome stain, ×20).

Microscopic evaluation was performed using Masson’s trichrome stain to clearly differentiate each layer. We first measured the tissue diameter after fixation. We next measured the full thickness of the fixed tissue, and then measured the submucosal depth. A total of five submucosal sites were measured to obtain a mean value (Fig 7).

### Statistical Analysis

Statistical analysis was performed using IBM SPSS Statistics for Windows, Version 20.0 (IBM Corp., Armonk, NY, USA) and R version 2.11.1 (R Core Group, Vienna, Austria). Data were expressed as means and standard deviations. The Fligner-Killeen test was applied to data that did not follow a normal distribution to test for the homogeneity of variances. The Mann-Whitney U test was used to test differences between medians. In all tests, P values < 0.05 were considered statistically significant.

## Results

### Calculation of the elastic modulus of tissue

In this study, a fixation device was developed to ensure constant tissue sample elasticity to prevent deformation during fixation. To validate this device, tissue elasticity was evaluated by measuring the elastic modulus of the tissue. In order to measure the tissue modulus independently from the tissue dimension, we used the equation shown in Formula 1 to calculate the elastic modulus of the tissues tested in this experiment. There was no significant elastic modulus difference among the 2, 3, and 4 cm tissue samples after fixation. (Table 1).

**Table 1:** Elastic modulus obtained at yield strength.

	2 cm	3 cm	4 cm
**Avg**	101.2	110.4	105.5
**Std**	175	149	164

Abbreviations: Avg, average; Std, standard deviation.

$$\text{E}=\frac{tensile\;stress}{tensile\;strain}=\frac{\sigma }{\varepsilon }=\frac{F/A_{0}}{\Delta L/L_{0}}=\frac{FL_{0}}{A_{0}\Delta L}$$ (1)

Formula 1. Young’s modulus

Abbreviations: E, elasticity; F, force exerted on a tissue under tension; A_0_, original cross-sectional area through which the force is applied; ΔL, amount by which the length of the tissue changes; L_0_, original length of the tissue

### Measurement of tissue eccentricity (ellipse) for all sample diameters

Each circular tissue sample was punched and fixed. However, during fixation the tissue can become deformed, which may affect fixation quality. Therefore, eccentricity was used as a measure to express the magnitude of tissue deformation. The tissue eccentricity was strictly less than 1 (Formula 2). A circle of tissue has an eccentricity close to 0. Values close to zero, indicate less tissue deformation. As shown in Table 2, the eccentricity of samples processed using the fixation device was similar to the control, suggesting that the device might result in less deformation of tissue to be fixed, an advantage for use in practical examinations.

**Table 2:** Comparison of eccentricity between fixation methods.

	Control	Manual pinning	Fixation device
**2 cm tissue**			
**Avg**	0.45	0.48	0.45
**Std**	0.11	0.12	0.14
**3 cm tissue**			
**Avg**	0.46	0.54	0.40
**Std**	0.1	0.13	0.10
**4 cm tissue**			
**Avg**	0.45	0.52	0.43
**Std**	0.10	0.12	0.15

Abbreviations: Avg, average; Std, standard deviation.

$$\text{e}=\sqrt{\left(1-\frac{b^{2}}{a^{2}}\right)}$$ (2)

Formula 2. Tissue eccentricity

Abbreviations: e, eccentricity; a, length of the semi-major axis; b, length of the semi-minor axis.

### Comparison of fixation methods for 2 cm tissue samples

We first measured the diameter and submucosal depth of the 2 cm diameter tissue samples. We calculated the statistical significance of each measure. While there were significant differences in the median measure of specimen diameter between the two groups, the differences in submucosal depth were not (Table 3). We focused on the difference of variances between fixation methods, statistical analysis using R 2.11.1 and Fligner-Killeen tests were used to measure these differences. The variance of the standard deviation was checked to confirm the homogeneity of the submucosal variance, with a resulting P-value of 0.012. The manual pinning group showed greater variation in submucosal thickness than that in fixation device group.

**Table 3:** Comparison of fixation methods.

	Diameter (mm)			Submucosal depth (μm)		
	Fixation device	Manual pinning	P value	Fixation device	Manual pinning	P value
**2 cm**						
**Med**	23.85	21.0	0.001	397.09	393.76	0.756
**Std**	0.98	1.74	0.206	84.40	179.47	0.012
**3 cm**						
**Med**	32.30	32.0	0.281	381.43	529.69	0.029
**Std**	1.73	2.16	0.573	102.51	203.23	0.042
**4 cm**						
**Med**	45.0	44.50	0.288	415.51	603.82	0.004
**Std**	1.41	2.09	0.897	70.79	284.55	0.001

Abbreviations: Med, median; Std, standard deviation.

### Comparison of fixation methods for 3 cm tissue samples

Among 3 cm tissues, the median submucosal depth differed significantly between the fixation device and pinning method groups. The fixation device resulted in the least submucosal depth variance, with a significant homogeneity of variance (0.042) in measurement of submucosal depth in the 3 cm tissue samples.

### Comparison of fixation methods for 4 cm tissue samples

Analysis of the 4 cm tissue samples revealed significant differences in the median submucosal depth between the two groups. Similar significant results were also seen in the test for homogeneity of variances (P = 0.001).

## Discussion

In the present study, we compared the variability of submucosal thickness between a novel tissue fixation device and conventional manual fixation. The fixation device was developed to ensure constant tissue elasticity in order to prevent tissue deformation during fixation. Rather than evaluating the differences between mean measures, this study determined whether the fixation device minimalized variation between resected specimens. The submucosal depth was much more consistent in the tissue fixation group compared to that in the manual fixation group, for all tissue diameters (2, 3, and 4 cm).

ESD is an effective endoscopic treatment developed to remove superficial gastrointestinal tract neoplasms [1,2]. The expanded criteria of ESD for EGC that included pathologic criteria, such as less than 500 μm invasion into the submucosal layer, were verified to have negligible risk of lymph node metastasis and usually resulted in excellent outcomes after endoscopic submucosal dissection [7,11–15]. However, several cases of local recurrence and distant metastasis, despite curative endoscopic resection of EGC according to the expanded indications, have been reported [7,8]. There are several possible explanations for these observations, including lymphovascular invasion or submucosal invasion more than 500 μm apart from the resected tissue margin. Second, differences in measurement methods to estimate invasion depth may contribute to such discrepancies [16]. Finally, human error during specimen handling may also lead to discrepancies in the evaluation of invasion depth and specimen size [9,10,17].

Tumor size and invasion depth, which could be affected by the force used to extend the resected ESD specimen during manual pinning, are important factors used to determine the further treatment plan based on the potential for lymph node metastasis. Recommendations suggest that physicians should not attempt to flatten ESD specimens with powerful stretching [9]. However, there are currently no standard recommendations for optimal manual tissue fixation methods.

To overcome this limitation, we invented a novel tissue fixation device to offer more objective and standardized tissue preparation.

Accurate evaluation of the depth of submucosal invasion in EGC samples after endoscopic treatment is important in order to determine further treatment plans. Because metastasis after curative treatment of gastric mucosal cancers may occur, further investigation of this issue is particularly important.

We assumed that human errors could occur during manual fixing of specimens to corkboard following ESD. These errors depend on individual stretching force applied by individual physicians, which could consequently result in inaccuracies in determining tumor size and depth of submucosal invasion. An objective method to fix resected tissue specimens before formalin fixation is essential for accurate pathologic assessment. However, current tissue fixation methods are more likely to be non-objective because of inconsistent stretching errors introduced by endoscopists responsible for fixing ESD specimens [18]. Because these unavoidable human errors could affect the measurement of submucosal invasion depth and resected specimen size, which in turn affect patient management or even the subsequent surgical procedures performed, more objective and consistent fixation methods are necessary for accurate pathologic assessment.

In this study, we designed a novel tissue fixation device and validated it by comparing the resulting submucosal thickness with that of manual tissue fixation on corkboard in a variety of resected tissue sizes. Analysis of the variance of the standard deviations showed that the submucosal thickness in the manual fixation group was much more variable than in the fixation device group. This method could reduce human error during manual fixation of ESD specimens.

This study has several limitations, including the use of porcine gastric tissues comprising a whole layer containing the thick muscular layer with less elastic features. This characteristic is different from specimens obtained after ESD, which usually consist of partial submucosa and entire mucosa that stretch more easily.

However, to accurately evaluate submucosal depth, measurement of the full thickness of the stomach wall is required. Although this device is a prototype and requires further development, it offers the potential to significantly reduce human error in tissue fixation after ESD. There are currently no standard criteria for ESD specimen fixation; further discussion is necessary to establish these guidelines. Further studies are also necessary to investigate human tissue and address potential drawbacks.

In conclusion, submucosal thickness using the novel fixation device was relatively consistent compared to conventional fixation. This fixation device may provide a more objective and convincing method for evaluation of submucosal invasion depth in ESD specimens.

## Supporting Information

S1 Video Tissue fixation device. (AVI)
